# The human intestinal microbiota of constipated-predominant irritable bowel syndrome patients exhibits anti-inflammatory properties

**DOI:** 10.1038/srep39399

**Published:** 2016-12-16

**Authors:** Alain P. Gobert, Giulia Sagrestani, Eve Delmas, Keith T. Wilson, Thomas G. Verriere, Michel Dapoigny, Christophe Del’homme, Annick Bernalier-Donadille

**Affiliations:** 1 Institut National de la Recherche Agronomique (INRA), UR454 Microbiologie, Saint-Genès-Champanelle, France; 2 Division of Gastroenterology, Hepatology, and Nutrition, Department of Medicine, Vanderbilt University Medical Center, Nashville, Tennessee, USA; 3 Center for Mucosal Inflammation and Cancer, Vanderbilt University Medical Center, Nashville, Tennessee, USA; 4 Department of Pathology, Microbiology, and Immunology, Vanderbilt University Medical Center, Nashville, Tennessee, USA; 5 Department of Cancer Biology, Vanderbilt University Medical Center, Nashville, Tennessee, USA; 6 Veterans Affairs Tennessee Valley Healthcare System, Nashville, Tennessee, USA; 7 CHU Clermont-Ferrand, Service de Médecine Digestive et Hépatobiliaire, Hôpital Estaing, Clermont-Ferrand, France

## Abstract

The intestinal microbiota of patients with constipated-predominant irritable bowel syndrome (C-IBS) displays chronic dysbiosis. Our aim was to determine whether this microbial imbalance instigates perturbation of the host intestinal mucosal immune response, using a model of human microbiota-associated rats (HMAR) and dextran sulfate sodium (DSS)-induced experimental colitis. The analysis of the microbiota composition revealed a decrease of the relative abundance of *Bacteroides, Roseburia-Eubacterium rectale* and *Bifidobacterium* and an increase of *Enterobacteriaceae, Desulfovibrio* sp., and mainly *Akkermansia muciniphila* in C-IBS patients compared to healthy individuals. The bacterial diversity of the gut microbiota of healthy individuals or C-IBS patients was maintained in corresponding HMAR. Animals harboring a C-IBS microbiota had reduced DSS colitis with a decreased expression of pro-inflammatory cytokines from innate, Th1, and Th17 responses. The pre-treatment of conventional C57BL/6 mice or HMAR with *A. muciniphila*, but not with *Escherichia coli*, prior exposure to DSS also resulted in a reduction of colitis severity, highlighting that the anti-inflammatory effect of the gut microbiota of C-IBS patients is mediated, in part, by *A. muciniphila*. This work highlights a novel aspect of the crosstalk between the gut microbiota of C-IBS patients and host intestinal homeostasis.

Irritable bowel syndrome (IBS) is a chronic functional disorder of the gastrointestinal tract that affects approximately 10% of the population worldwide^1^. IBS has a critical impact on the quality of life of patients and represents the most frequent reason for referral to gastroenterology outpatient clinics. Despite the absence of intestinal structural abnormality, IBS patients experience recurrent abdominal pain, bloating, and altered bowel habits, with constipation, diarrhea, or both^2^. Although the precise etiology of the pathophysiological changes underlying IBS development remains unclear, substantial evidence indicates that dysfunction in the bidirectional interactions between the intestine and the nervous system has an important role in the symptomatology of IBS^3^. In this context, it has been demonstrated that composition and/or function of the intestinal microbiota, which is an essential armature for intestinal homeostasis and influences central nervous system function^4^, is altered in IBS patients^5^,^6^, and may therefore play a key role in the pathogenesis of this disease. Of importance, we have recently demonstrated using human microbiota-associated rats (HMAR) that the functional dysbiosis of the gut microbiota of constipated IBS (C-IBS) patients can induce visceral hypersensitivity^7^.

Recent theories on the pathophysiology of IBS have also integrated interactions between neural and immunologic networks within the intestinal wall. Clinical studies have emphasized an increased number of mast cells throughout the intestinal tract of IBS patients compared with healthy volunteers, notably those in the vicinity of visceral neurons^8^. Further, it has been described that the main mast cell degranulation products, i.e. tryptase, histamine, and serotonin, are involved in activation of submucosal neurons^9^,^10^,^11^ and may therefore participate in visceral hypersensitivity and abdominal pain in IBS patients. However, the recruitment and the role in IBS pathogenesis of other cells of the innate response and of cellular immunity, which could be implicated in a low grade inflammatory state of the intestinal mucosa, are still unclear. Notwithstanding the particular case of post-infectious IBS and patients in remission from inflammatory bowel disease^12^, there is no consensus about the activation and the role of the mucosal immune system in this functional bowel disorder. Examination of pro- and anti-inflammatory cytokines in the gut or in the serum of IBS patients has led to conflicting results^13^,^14^,^15^. Similarly, while low grade infiltration of T cells has been observed in the lamina propria^16^,^17^ or in the myenteric plexus^18^ of IBS patients, numerous studies has also found normal or decreased lymphocyte density in the intestinal tissues^19^,^20^.

In this context, our aim was to investigate the effect of the human intestinal microbiota of C-IBS patients on the mucosal immune response. For this purpose, we used HMAR, and demonstrate that the gut microbiota of C-IBS patients protects animals from dextran sulfate sodium salt (DSS)-induced colitis. This protective effect is mediated by the increased abundance of the bacterium *Akkermensia muciniphila* in the C-IBS microbiota compared to healthy subjects.

## Results

### Composition and diversity of fecal microbiota from healthy and C-IBS subjects and from HMAR

We first analyzed the genetic bacterial diversity between the microbiota of healthy and C-IBS individuals and their corresponding HMAR. Pyrosequencing analysis performed on human and HMAR fecal DNA generated an average of 11,513 ± 4,223 high quality, taxonomically classifiable 16S rDNA gene sequences with mean read lengths of 253.3 ± 2.7 nt. Richness and diversity of fecal microbiota remained very similar between the human intestinal microbiota of healthy subjects and C-IBS patients, and the respective HMAR (Supplementary Table S1).

**Figure 1:**
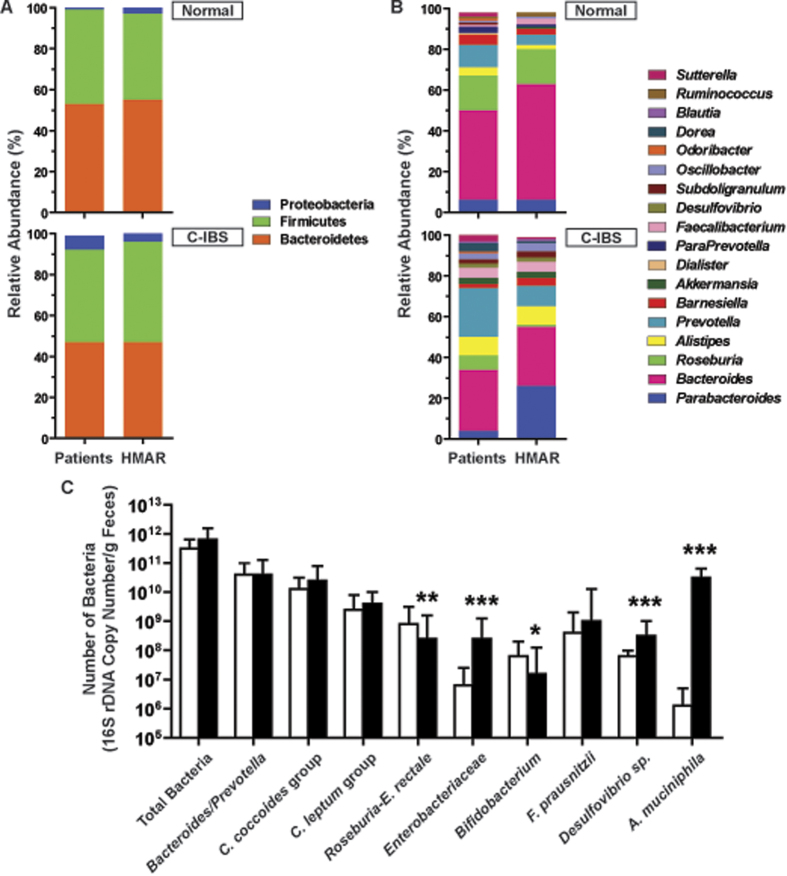
Comparison of bacterial composition and diversity in fecal samples from human and HMAR. Relative 16S rDNA gene abundances of the major phyla (**A**) and genera (**B**) detected in human and HMAR fecal samples. These figures represent the data obtained with the fecal microbiota of one healthy individual and one C-IBS patient, and of the corresponding H-MAR and C-IBS HMAR; similar data have been obtained with the two others healthy individuals and C-IBS patients. In (**A**), there was no significant differences between the composition of the microbiota of the human subjects and HMAR: Healthy microbiota, *p* = 0.54; C-IBS microbiota, *p* = 0.35. (**C**) Quantification of the major gut bacterial populations or species in healthy (open bars) or C-IBS microbiota (plain bars). The figure depicts the quantitative PCR data obtained with the fecal samples of HMAR derived from one healthy individual and one C-IBS patient; *n* = 5 rats for healthy microbiota and *n* = 5 rats for C-IBS microbiota. *P < 0.05, **P < 0.01, ***P < 0.001, denote significant difference vs. N-HMAR.

At the phylum level, 454-pyrosequencing analysis showed that the fecal microbiota of human IBS and healthy subjects were dominated by the *Firmicutes* and *Bacteroidetes* at similar relative abundance, and C-IBS patients harboured more *Proteobacteria* than healthy ones (*p* = 0.033; Fig. 1A). The relative abundance of the *Firmicutes, Bacteroidetes* and *Proteobacteria* phyla was similar in the fecal samples of human and HMAR (Fig. 1A).

At the genus level, the most abundant bacterial genera detected by pyrosequencing were *Bacteroides, Prevotella* and *Roseburia* in both healthy and C-IBS subjects, *Prevotella* being more abundant and *Roseburia* less present in C-IBS subjects compared to healthy ones (*p* = 0.0045; Fig. 1B). A similar dysbiosis was observed between N-HMAR and C-IBS-HMAR (*p* = 0.0011; Fig. 1B). Moreover, we found increased abundance of *Alistipes, Desulfovibrio*, and *Akkermensia* in the gut microbiota of C-IBS patients and C-IBS-HMAR than in those of healthy subjects and N-HMAR, respectively (Fig. 1B). Although, the relative abundance of *Prevotella* was reduced and that of *Parabacteroides* was increased in the gut microbiota of C-IBS-HMAR in comparison with C-IBS patients (*p* < 0.0001; Fig. 1B), these data collectively demonstrate that the bacterial diversity of the intestinal microbiota of healthy subjects and C-IBS patients was maintained in the corresponding HMAR.

Then, we demonstrated by qPCR that there was less *Bifidobacterium* and bacteria from the *Roseburia*-*E. rectale* group in C-IBS-HMAR than in N-HMAR (Fig. 1C). In contrast, we found more *Enterobacteriaceae, Desulfovibrio*, and *A. muciniphila* in the gut microbiota of C-IBS-HMAR than in that of N-HMAR (Fig. 1C).

We decided to confirm the observation concerning *A. muciniphila* population level in C-IBS on a large number of patients, and found that this bacterial species was significantly more prevalent in the fecal microbiota of C-IBS patients in comparison with healthy individuals (Supplementary Fig. S1).

### The gut microbiota from C-IBS patients protects from DSS-induced colitis

**Figure 2:**
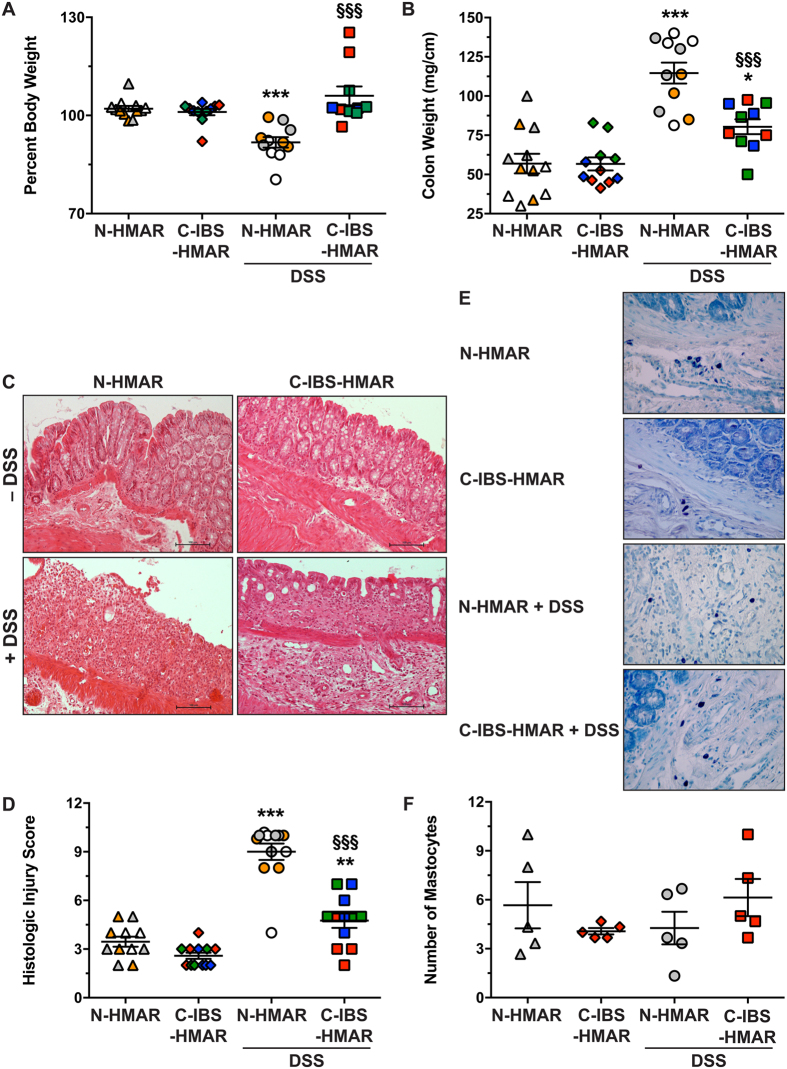
Effect of healthy and C-IBS microbiota on colonic inflammation. N-HMAR and C-IBS-HMAR were given water or 4% DSS in their drinking water for 7 days. (A) Body weights of the rats were measured before DSS treatment and at the end of the experiment, and are presented as percentage of initial body weight. (**B**) Colon weights and lengths were measured and the ratio is presented for each animal. (**C and D**) Colons were fixed and stained with hematoxylin-eosin-saffron; representative photomicrographs of N-HMA and C-IBS-HMA rat colons treated or not with DSS are shown (**C**); these tissues were scored for total histological injury (**D**). For A, B, and D, **P < 0.01, ***P < 0.001 vs. N-HMA or C-IBS-HMA without DSS; ^§§§^*P* < 0.001 vs. N-HMAR treated with DSS. The data depict experiments performed with the gut microbiota of three different control or C-IBS patients inoculated to 3–5 rats; each symbol represents one animal and each color depicts the microbiota of one individual. (**E and F**) Representative staining of colons for mastocytes (dark violet; **E**); the mean of the number of mastocytes observed in three fields (X400) for each tissue is presented in F. The data obtained with the microbiota of one healthy individual and one C-IBS subject is shown.

Rats with a human microbiota from healthy or C-IBS patients were treated or not with DSS for 7 days to induce colitis (Supplementary Fig. S2). We did not observe mortality in both groups of HMAR receiving DSS. However, N-HMAR significantly lost weight under DSS treatment when compared to untreated animals, whereas DSS had no effect on weight loss in C-IBS-HMAR (Fig. 2A). Moreover, colon weight-to-length ratio was significantly increased in both groups of rats treated with DSS, but was greater by ~13.5% in N-HMAR than in C-IBS-HMAR (Fig. 2B).

Histological sections revealed colonic inflammation and epithelial damage in DSS-treated rats (Fig. 2C). Edema, crypt abscesses, and congestion of the lamina propria were more marked in rats harboring the human microbiota from healthy subjects compared to C-IBS-HMAR (Fig. 2C). Using a comprehensive scoring system to quantify the degree of damage, we confirmed that DSS treatment led to increased histological injury in N-HMAR (Fig. 2D) compared to C-IBS-HMAR. There was no detectable inflammation or epithelial injury in untreated HMAR (Fig. 2C and D).

Because increased mastocyte infiltration has been observed in the colon of IBS patients, we assessed this parameter in HMAR. We found no significant differences between animals harboring healthy or C-IBS gut microbiota, and DSS had no effect on recruitment of mastocytes in the colonic tissues (Fig. 2E and F).

### Decreased colonic immune response in rats exhibiting microbiota from C-IBS patients

**Figure 3:**
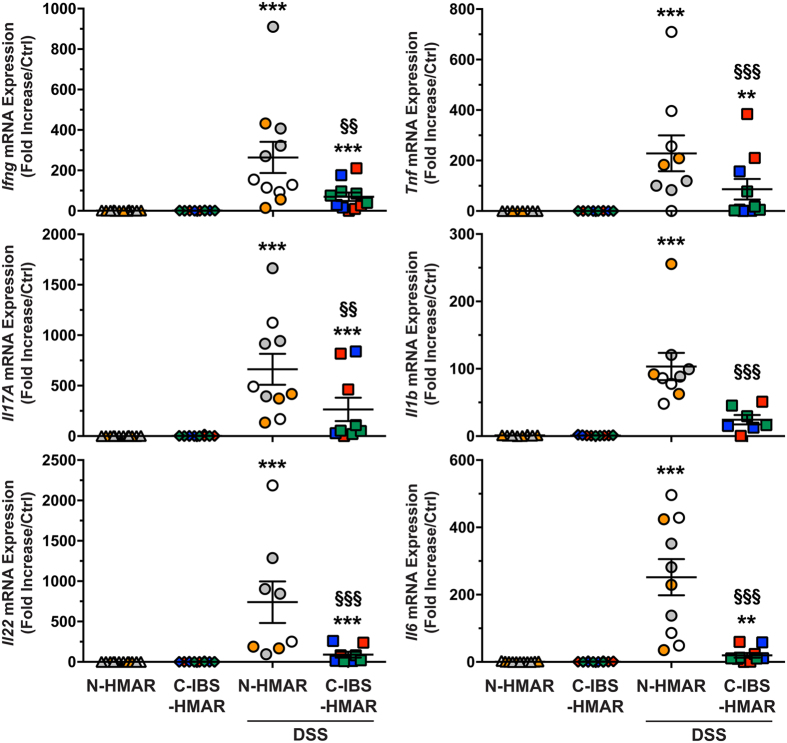
Effect of normal and C-IBS fecal microbiota on cytokine levels. Expression level of the genes encoding IFN-γ, TNF-α, IL-17, IL-1β, IL-22, and IL-6 in the colons of N-HMAR or C-IBS-HMAR, treated or not with DSS. **P < 0.01, ***P < 0.001 vs. untreated rats; ^§^*P* < 0.05, ^§§^*P* < 0.01, ^§§§^*P* < 0.001 compared to N-HMAR receiving DSS. These data represent experiments performed with the gut microbiota of three different control or C-IBS patients inoculated to 3–5 rats. Each symbol represents one animal and each color depicts the microbiota of one individual.

To further assess the role of the intestinal microbiota on the immune response during colitis, we analyzed the expression of the genes encoding mediators of the pro-inflammatory response. As shown in Fig. 3, the treatment of the rats harboring a human intestinal microbiota by DSS led to an increased expression of the genes encoding IFN-γ, IL-17, and IL-22, the prototype cytokines of Th1, Th17, and Th17/Th22 responses, as well as those from the innate immune system, namely TNF-α, IL-1β, and IL-6, compared to untreated rats. It is noteworthy that there were significantly more transcripts of all these pro-inflammatory genes in colonic tissues from DSS-treated N-HMAR than in those from animals with an intestinal microbiota from C-IBS patients (Fig. 3).

### Pre-treatment of animals with *A. muciniphila* protects from colitis

Quantification of *A. muciniphila*, a bacterial species from the human gut microbiota that exhibits anti-inflammatory properties^21^,^22^, revealed that C-IBS-HMAR harbored higher *A. muciniphila* population than N-HMAR (Fig. 1C). We therefore reasoned that this bacteria might play a role in protection of C-IBS-HMAR during DSS colitis. To first test this hypothesis in an well-established colitis model, we inoculated *A. muciniphila* or the commensal human strain, *E. coli* EcG2, to C57BL/6 mice for 15 days before the treatment with DSS.

**Figure 4:**
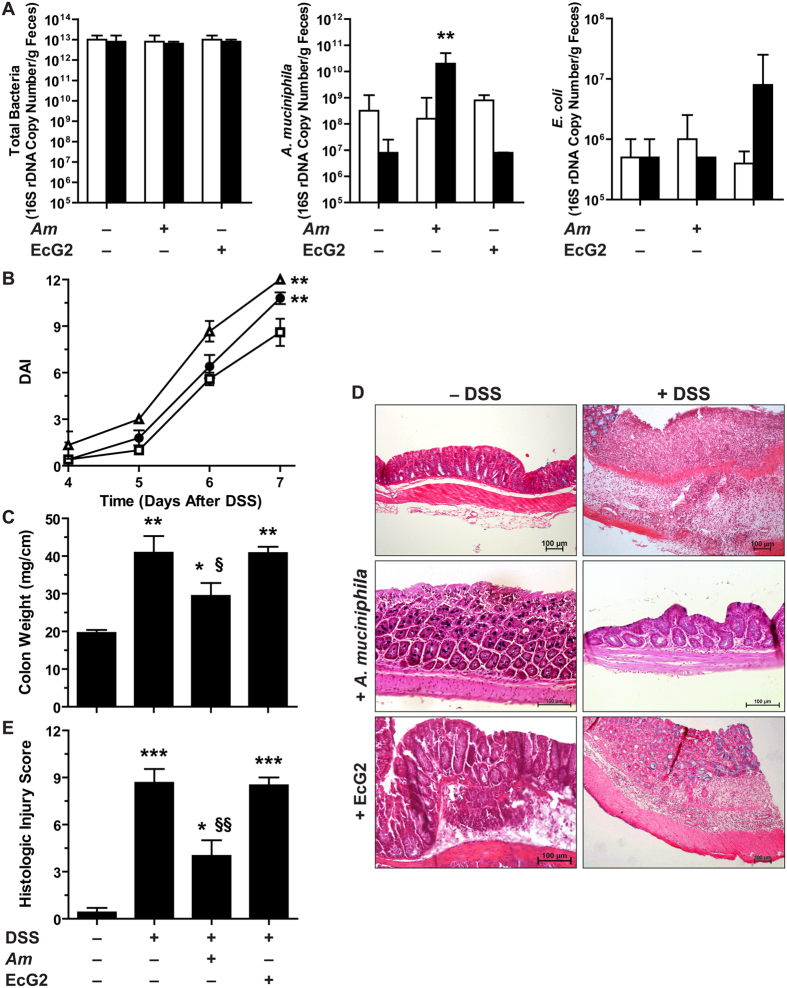
Colitis in mice pre-treated with *A. muciniphila* or *E.coli*. (**A**) C57BL/6 mice were given daily PBS, *A. muciniphila* (*Am*), or *E.coli* EcG2 (5 × 10^8^/mice in 100 μl for both bacteria) for 15 days. The number of total bacteria, *A. muciniphila* and *E. coli* in the feces was determined by qPCR before (open bars) or after the treatment (plain bars). **P < 0.01 compared to the same group before treatment with bacteria; *n* = 3 mice per group. (**B**) Mice pre-treated with *A. muciniphila* (open squares), *E.coli* EcG2 (open triangles), or with vehicle (plain circles) were given 4% DSS and were monitored daily. **P < 0.01 vs mice + *A. muciniphila*; *n* = 5 mice not treated with DSS and 10 mice per group for the DSS-treated mice. The DAI of animals pre-treated or not with *A. muciniphila* or *E. coli* but not receiving DSS was 0–1. (**C–E**) After 7 days of DSS, mice were euthanatized and colon weight-to-length ratios (**C**) and histological parameters (**D and E**) were analyzed. *P < 0.05, **P < 0.01, ***P < 0.001 vs. Ctrl; ^§^*P* < 0.05, ^§§^*P* < 0.01 vs. animals receiving DSS only or pre-treated with EcG2; *n* = 5 mice not treated with DSS and 10 mice per group for the DSS-treated mice.

The total number of bacteria in the fecal content of mice was not affected by the administration of *A. muciniphila* or *E. coli* (Fig. 4A). The population level of *A. muciniphila* was increased by 2-log orders in feces of animals that received this bacterium for 15 days (Fig. 4A). In a similar way, the administration of EcG2 led to an increased number of *E. coli* (Fig. 4A).

The disease activity index (DAI; Fig. 4B) and colon weight-to-length ratio (Fig. 4C) were increased in mice treated with DSS compared to untreated animals; these parameters were significantly reduced when mice received *A. muciniphila*, but not *E. coli* EcG2 (Fig. 4B and C). A severe colitis showing mucosal and submucosal infiltration with mononuclear cells, associated with epithelial erosion, crypt effacement, and edema, was observed in mice treated with DSS (Fig. 4E and D). Inflammation and epithelial injury were significantly less marked in animals pre-treated with *A. muciniphila* (Fig. 4E and D). The administration of *A. muciniphila* or EcG2 had no impact on mice not treated with DSS (Fig. 4D).

**Figure 5:**
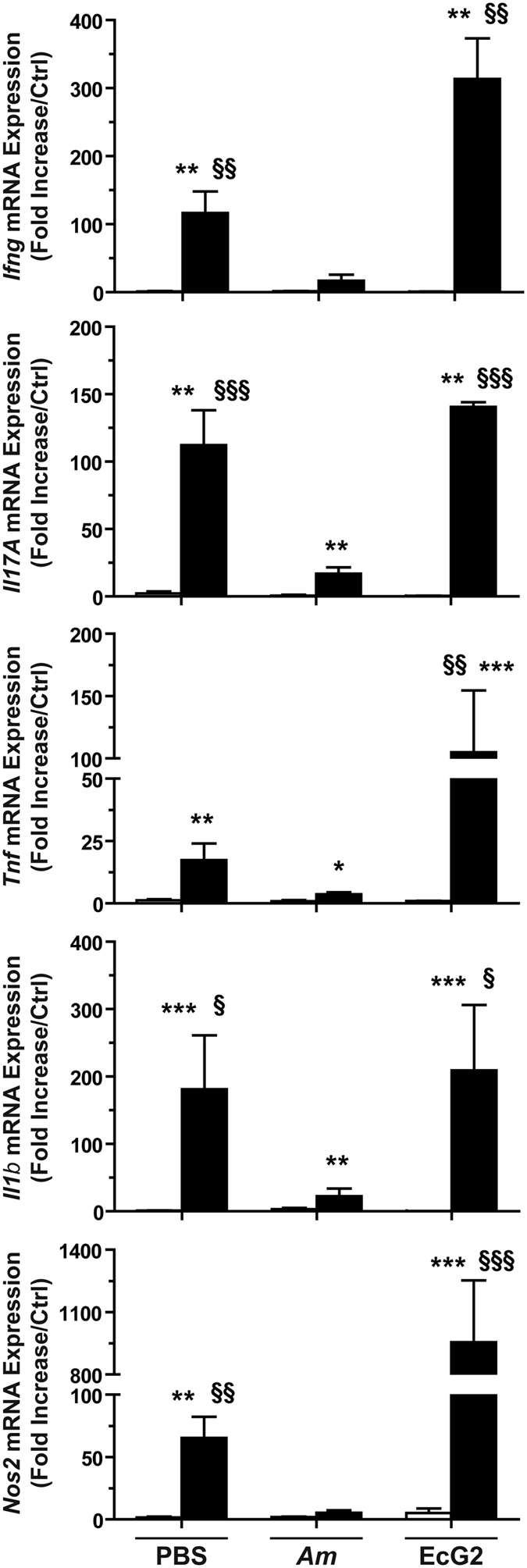
Expression of the genes encoding pro-inflammatory effectors in mice. Animals were given *A. muciniphila* (*Am*), *E. coli* EcG2, or the vehicle (PBS) for 15 days, and then they were treated (plain bars) or not (open bars) with DSS. The expression of the genes encoding IFN-γ, TNF-α, IL-17, IL-1β, and NOS2 in the colons were analyzed by RT-qPCR. *P < 0.05, **P < 0.01, ***P < 0.001 vs. not treated with DSS; ^§^*P* < 0.05, ^§§^*P* < 0.01, ^§§§^*P* < 0.001 compared to animals pre-treated with *A. muciniphila* and receiving DSS; *n* = 10 mice per group.

In addition, the DSS-induced expression of the genes encoding the pro-inflammatory effectors IFN-γ, IL-17, TNF-α, IL-1β, and NOS2 was repressed in mice pre-treated with *A. muciniphila* when compared to animals that have received the vehicle or *E. coli* EcG2 (Fig. 5).

**Figure 6:**
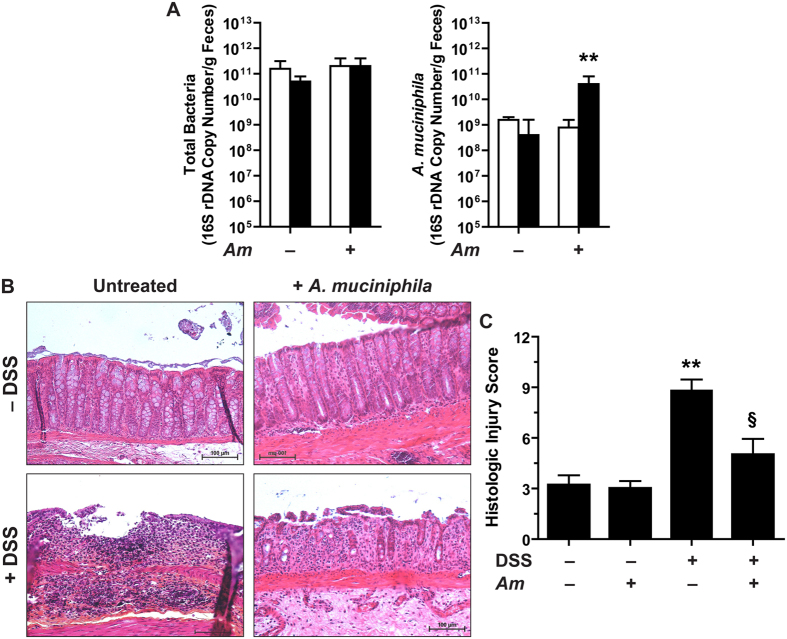
Effect of *A. muciniphila* on colitis in HMAR (**A**) N-HMAR were treated daily for 15 days with 5 × 10^9^ *A. muciniphila* (*Am*) or with PBS; quantification of total bacteria and *A. muciniphila* was performed by qPCR before (open bars) or after the treatment (plain bars). **P < 0.01 compared to the same group before treatment with *A. muciniphila*; *n* = 5 N-HMAR in each group. (**B**) N-HMAR inoculated or not with *A. muciniphila* were then exposed to water or to 4% DSS for 7 days and euthanatized. Colons were harvested and stained; representative photomicrographs of each group of animals are shown. (**C**) Colons were scored for total histological injury. **P < 0.01 vs. N-HMAR without DSS; ^§^*P* < 0.05 vs. animals treated with DSS; *n* = 5 N-HMAR in each group.

We next verified that *A. muciniphila* also exerts a protective effect in the presence of a human intestinal microbiota. The population of *A. muciniphila* in the feces was increased by 2 log in animals treated daily with this bacterial species for a 15 days period, while the total number of bacteria was not modified (Fig. 6A). N-HMAR pre-treated with *A. muciniphila* and receiving DSS exhibited significantly less histologic damages than animals receiving DSS only (Fig. 6B and C). The treatment with *A. muciniphila* in rats without DSS had no effect on histologic parameters (Fig. 6B and C).

## Discussion

The current study demonstrates that the intestinal microbiota of C-IBS patients protects from DSS-induced colitis. The essential role of *A. muciniphila*, which is more prevalent in gut microbiota of these patients than in healthy individuals, in this protective effect was supported by our findings that mice and HMAR pre-treated with this bacteria exhibit reduced histological damages in the colon and reduced tissue mRNA expression of pro-inflammatory mediators of the innate, Th1 and Th17 responses.

Previous work from our group demonstrated that the transfer of the intestinal microbiota from C-IBS patients to germ-free (GF) rats leads to an increase in visceral sensitivity compared to N-HMAR or to conventional animals^7^. This suggests that the functional dysbiosis of the C-IBS microbiota^6^ may affect the intestinal host response. In addition to this feature, we now demonstrated that the intestinal microbiota of C-IBS patients displays anti-inflammatory properties during experimental colitis. This result is somehow unexpected since histopathological investigations have evidenced a microscopic inflammation in the colon of IBS patients, mainly characterized by an augmentation of mast cells, intraepithelial lymphocytes and CD3^+^ T cells in the colonic mucosa^8^,^16^. Nonetheless, other have shown no evidence of activation of mucosal or pervasive immune responses compared to normal individuals^20^,^23^ or an increased infiltration of anti-inflammatory cells such as CD25^+^ T lymphocytes^16^. In the same way, we did not observe significant changes in mast cell infiltration, or histologic and inflammatory parameters of the colon in N-HMAR or C-IBS-HMAR, not treated with DSS, as previously described^7^.

The role of the gut microbiota of IBS patients in the establishment of a potential low-grade mucosal immune response has not been previously determined. Rather, numerous studies have highlighted that patients with inflammatory bowel diseases (IBD) and IBS exhibit dissimilar intestinal microbiota dysbiosis. Compared to healthy individuals, the *Bifidobacterium, Ruminoccoccus* and *Lactobacillus* genus are increased in IBD^24^,^25^ and decreased in IBS^6^,^26^,^27^; moreover, the IBD gut microbiota shows a lower level of *F. prausnitzii* and a higher level of *E. coli* compared to IBS microbiota^24^,^28^. Of particular interest, while we show herein for the first time that *A. muciniphila* is significantly increased in the intestinal microbiota of C-IBS patients, which is consistent with the higher prevalence of this bacterium in slow transit individuals^29^, it has been reported that its abundance is reduced in the mucosa of patients with Crohn’s disease or ulcerative colitis^30^,^31^. Although IBS and IBD microbiota were not directly analyze in head to head comparison and that different methodological approaches were used, these findings support the evidence that *A. muciniphila* is an essential commensal bacteria that may explain the anti-inflammatory properties of the C-IBS microbiota. Importantly, the protective effect of the C-IBS gut microbiota on colitis is not likely due to *F. prausnitzii*, another bacteria of the human microbiota presenting anti-inflammatory properties^32^, because its population level was similar in normal and C-IBS microbiota, as reported^28^, as well as in N-HMAR and C-IBS-HMAR.

*A. muciniphila* is a bacterium that adheres to intestinal epithelial cells^33^ and may use the mucus as a sole source of carbon and nitrogen^34^. The decreased abundance of this bacterium has been correlated with type 2 diabetes, obesity and metabolic syndrome^35^,^36^. Moreover, animal models have emphasized that supplementation with *A. muciniphila* limits body fat accumulation and prevents endotoxemia and infiltration of myeloid cells in adipose tissues in diet-induced obese mice^35^. Similarly, it has been shown that the increase of the proportion of *A. muciniphila* in the gut microbiota using polyphenols reduces NF-κB activation and TNF-α production in the intestine of mice fed a high fat diet^22^. These investigations support our finding that *A. muciniphila* possesses anti-inflammatory properties and thus protects against colitis. Further, the *A. muciniphila*-dependent strengthening of the intestinal barrier^33^, the regulation of the immune response by the outer membrane vesicles of *A. muciniphila*^21^, and/or the production of short-chain fatty acids including propionate and acetate^34^ that have been shown to display anti-inflammatory effects through G protein-coupled receptor signaling^37^,^38^, may provide a rationale to explain the protective function of this bacterium against colitis. However, further investigations are warranted to delineate the cellular mechanisms by which *A. muciniphila* dampens DSS-induced inflammatory processes. It is also critical to note that, although an overall increased prevalence of *A. muciniphila* has been evidenced in C-IBS microbiota compared to healthy patients, some C-IBS and healthy patients display low and high prevalence of the bacterium, respectively. Therefore, our finding that the C-IBS microbiota protect from colitis cannot be generalized to all C-IBS subjects. Rather, we suggest that the *A. muciniphila*^high^ microbiota exhibits anti-inflammatory properties, but further investigations are mandatory to confirm this hypothesis.

While patients with IBD may experience functional bowel symptoms^39^, there is no clear evidence that IBS individuals are predisposed or protected against chronic intestinal inflammation. It would be highly speculative to conclude from our data that IBS patients are protected against IBD since their etiology is multifactorial and may include genetic, immunologic, microbiologic, and environmental factors^40^. However, we provide evidences that the gut microbiota of C-IBS patients exhibits anti-inflammatory properties that can be attributed to the high prevalence of *A. muciniphila*. Therefore, we propose that the purported low-grade inflammation observed in C-IBS patients is not associated with dysbiosis of their intestinal microbiota.

## Methods

### Bacterial species

We used the human commensal *Escherichia coli* strain EcG2, isolated in our laboratory from feces of a healthy human^41^, and *A. muciniphila* (DSMZ 22959) obtained from the Deutsche Sammlung von Bakterien und Zellkulturen GmbH. *E. coli* was cultivated in LB medium while *A. muciniphila* was routinely maintained in a semi-synthetic anaerobic medium containing porcine stomach mucins^42^.

### IBS patients and healthy controls

The study protocol was designed in accordance to the French Comité de Protection des Personnes (CPP) and was approved by the Local Human Ethics Committee (CPP Sud-Est VI, France). Informed consent was obtained from all subjects for analyses of intestinal microbiota.

C-IBS patients (n = 33; 18–60 years old) included in the study fulfilled Rome III criteria^2^. Notably, subjects had three or less bowel movements per week. Healthy subjects (n = 58; 18–55 year-old) did not have gastrointestinal symptom and a normal stool frequency (one or two stools per day). Exclusion criteria included antibiotic therapy within the previous two months, organic intestinal disease, other systemic disease, previous gastro-intestinal surgery, inflammatory bowel disease, family history of colon cancer, treatment for depression, known psychiatric pathology, pregnancy, known allergy, alcohol or tobacco abuse (more than 30 g alcohol or 10 cigarettes per day). IBS subjects were advised not to take treatments with potential effects on gastrointestinal motor function as well as antispasmodic and/or antalgic during the week prior to fecal sampling. IBS and healthy subjects were further required to have a customary consumption of 10 to 20 g of dietary fibers, evaluated using a dietary questionnaire. All volunteers had a body mass index between 18 and 30 kg/m^2^.

For HMAR, three C-IBS patients (females; 45–50 years old) and three healthy subjects (females; 45–57 years old) were prospectively recruited in the Gastroenterology clinic of Estaing Hospital at Clermont-Ferrand, according to the criteria described above. Fecal samples from these subjects were collected under anaerobic conditions using Anaerocult A sachet (Merck) and processed within three hours.

### Animals, HMAR, and colitis

GF male Sprague Dawley rats were bred and housed in positive-pressure sterile isolators with free access to irradiated standard rodent diet (UAR, Villemoisson, France) and sterilized drinking water. At 8–10 weeks old, GF rats were inoculated *per os* with 2 ml of a 10^3^-fold dilution of human fecal samples from healthy subjects or C-IBS patients^7^. Each microbiota of healthy subjects or C-IBS patients was separately inoculated *per os* (2 ml of a 10^3^-fold dilution of human fecal samples) in GF rats aged 8–10 weeks. These animals were then fed with gamma irradiated humanized diet (U8958; Safe, Augy, France) for 4–6 weeks and used for two different protocols (Supplementary Fig. S2): 1/N-HMAR or C-IBS-HMAR were treated with 4% DSS (mol wt 36,000–50,000; MP Biomedicals) added to the drinking water; consumption of water and DSS was monitored daily and did not differ between N-HMAR or C-IBS-HMAR. 2/N-HMAR were given or not 5 × 10^8^ *A. muciniphila* in 1 ml PBS daily for 15 days before the treatment with 4% DSS. In both protocols, animals were euthanized 7 days after the treatment with DSS.

Specific pathogen free C57BL/6 mice (4 weeks old) were purchased from Janvier Labs. Mice were pre-treated or not with *A. muciniphila* 22959 or *E. coli* EcG2 (5 × 10^8^ bacteria/mice in 100 μl) each day for 15 days and then treated with 4% DSS in drinking water.

DAI was determined by scoring the extent of body weight loss, blood in the stool, and stool consistency^43^.

All experimental protocols were performed in accordance to the European Directive 2010/63/EU on the protection of animals used for scientific purposes and was approved by the local Institutional Animal Care and Use Committee of Auvergne, France.

### Fecal microbiota analysis

Fecal samples from C-IBS patients and healthy subjects, HMAR, and mice were homogenized with 3 mm glass beads in a bead beater (Retsch MM200) for 5 min at 1500 rpm; total DNA was then purified using the QIAamp DNA Stool Mini kit (Qiagen).

Pyrosequencing analysis was then performed by DNAVision SA using a 454 Life sciences Genome FLX instrument (Roche Applied Sciences). Resulting reads from the hypervariable gene region V5-V6 of the 16S rDNA gene were assigned to samples according to their multiplex identifier tag, checked for the presence of primer sequences and fragments length superior to 200 bp. All reads not fulfilling these criteria were discarded. Sequences were assigned to family and genus level using Mothur^44^ and Greengenes 16S reference database^45^. Chimera candidates were identified using the UCHIME implementation in Mothur^46^. Relative abundances of unassigned reads and reads assigned on family or genus level were calculated from the total number of reads matching the quality control criteria.

The main taxonomic groups or species of the gut microbiota were also quantified in fecal samples quantitative PCR. Total bacterial DNA (50 ng) was amplified using LightCycler FastStart DNA Master SYBR Green I (Roche) and specific 16S rDNA gene primers (Supplementary Table S2). Standard curves, obtained by serial 10-fold dilution of 16S rDNA gene purified using QIAquick PCR purification Kit (Qiagen) from the referent species.

### Histological injury scores

Colons were fixed in formalin, embedded in paraffin, and 5-μm sections were stained with hematoxylin-eosin-saffron and examined by two veterinary pathologists from the College of Veterinary Medicine, Food Science and Engineering (Nantes, France), who were blinded to the status of the tissues. The histologic score of colitis (0–12), which derived from the sum of the scores for inflammation extent, infiltration with inflammatory cells, damage in crypt architecture, edema, and presence of ulceration and/or crypt abscess, was determined as described^47^. The number of mastocytes was determined in each colonic section after toluidine blue staining, as reported^48^.

### Analysis of mRNA levels

RNAs from colon tissues were extracted using TRIzol Reagent (Life Technologies) and purified with lithium chloride as reported^49^. Subsequently, 1 μg RNAs from each sample were reverse-transcribed using oligo-dT primers and 5 U/μl of Superscript II reverse transcriptase (Invitrogen). cDNAs (1 μl) were amplified by the QuantiFast SYBR Green PCR Kit (Qiagen) and the primers described in Supplementary Table S2; the gene *Actb* that encodes β-actin was used as housekeeping gene. Results were expressed as relative mRNA expression compared to untreated GF rats or to control mice.

### Statistics

Figures and statistics were performed using the Prism 7.0a software. All the data shown represent the mean ± SEM. Chi-Square test was performed to compare the relative abundance of the bacterial phylum/genus of the intestinal microbiota. Data that were not normally distributed according to the D’Agostino & Pearson normality test were log transformed and distribution was re-assessed. Student’s t test or ANOVA with the Newman-Keuls test were used to determine significant differences between two groups or to analyze significant differences among multiple test groups, respectively.

## Additional Information

**How to cite this article**: Gobert, A. P. *et al*. The human intestinal microbiota of constipated-predominant irritable bowel syndrome patients exhibits anti-inflammatory properties. *Sci. Rep.* **6**, 39399; doi: 10.1038/srep39399 (2016).

**Publisher’s note:** Springer Nature remains neutral with regard to jurisdictional claims in published maps and institutional affiliations.

## Supplementary Material

Supplementary Information

## References

[b1] Canavan C., West J. & Card T. The epidemiology of irritable bowel syndrome. Clin Epidemiol 6, 71–80 (2014). 24523597 10.2147/CLEP.S40245 PMC3921083

[b2] Longstreth G. F. . Functional bowel disorders. Gastroenterology 130, 1480–1491 (2006). 16678561 10.1053/j.gastro.2005.11.061

[b3] Moloney R. D. . Stress and the Microbiota-Gut-Brain Axis in Visceral Pain: Relevance to Irritable Bowel Syndrome. CNS Neurosci Ther (2015). 10.1111/cns.12490 PMC6492884 26662472

[b4] Foster J. A., Lyte M., Meyer E. & Cryan J. F. Gut Microbiota and Brain Function: An Evolving Field in Neuroscience. Int J Neuropsychopharmacol (2015). 10.1093/ijnp/pyv114 PMC4886662 26438800

[b5] Rajilic-Stojanovic M. . Global and deep molecular analysis of microbiota signatures in fecal samples from patients with irritable bowel syndrome. Gastroenterology 141, 1792–1801 (2011). 21820992 10.1053/j.gastro.2011.07.043

[b6] Chassard C. . Functional dysbiosis within the gut microbiota of patients with constipated-irritable bowel syndrome. Aliment Pharmacol Ther 35, 828–838 (2012). 22315951 10.1111/j.1365-2036.2012.05007.x

[b7] Crouzet L. . The hypersensitivity to colonic distension of IBS patients can be transferred to rats through their fecal microbiota. Neurogastroenterol Motil 25, 12103 (2013). 10.1111/nmo.12103 23433203

[b8] Barbara G. . Activated mast cells in proximity to colonic nerves correlate with abdominal pain in irritable bowel syndrome. Gastroenterology 126, 693–702 (2004). 14988823 10.1053/j.gastro.2003.11.055

[b9] Barbara G. . Mast cell-dependent excitation of visceral-nociceptive sensory neurons in irritable bowel syndrome. Gastroenterology 132, 26–37 (2007). 17241857 10.1053/j.gastro.2006.11.039

[b10] Cenac N. . Role for protease activity in visceral pain in irritable bowel syndrome. J Clin Invest 117, 636–647 (2007). 17304351 10.1172/JCI29255 PMC1794118

[b11] Buhner S. . Activation of human enteric neurons by supernatants of colonic biopsy specimens from patients with irritable bowel syndrome. Gastroenterology 137, 1425–1434 (2009). 19596012 10.1053/j.gastro.2009.07.005

[b12] Collins S. M., Piche T. & Rampal P. The putative role of inflammation in the irritable bowel syndrome. Gut 49, 743–745 (2001). 11709500 10.1136/gut.49.6.743 PMC1728553

[b13] Kindt S. . Immune dysfunction in patients with functional gastrointestinal disorders. Neurogastroenterol Motil 21, 389–398 (2009). 19126184 10.1111/j.1365-2982.2008.01220.x

[b14] Chang L. . Serum and colonic mucosal immune markers in irritable bowel syndrome. Am J Gastroenterol 107, 262–272 (2012). 22158028 10.1038/ajg.2011.423 PMC3297737

[b15] Matricon J. . Review article: Associations between immune activation, intestinal permeability and the irritable bowel syndrome. Aliment Pharmacol Ther 36, 1009–1031 (2012). 23066886 10.1111/apt.12080

[b16] Chadwick V. S. . Activation of the mucosal immune system in irritable bowel syndrome. Gastroenterology 122, 1778–1783 (2002). 12055584 10.1053/gast.2002.33579

[b17] Cremon C. . Mucosal immune activation in irritable bowel syndrome: gender-dependence and association with digestive symptoms. Am J Gastroenterol 104, 392–400 (2009). 19174797 10.1038/ajg.2008.94

[b18] Tornblom H., Lindberg G., Nyberg B. & Veress B. Full-thickness biopsy of the jejunum reveals inflammation and enteric neuropathy in irritable bowel syndrome. Gastroenterology 123, 1972–1979 (2002). 12454854 10.1053/gast.2002.37059

[b19] Lee K. J. . The alteration of enterochromaffin cell, mast cell, and lamina propria T lymphocyte numbers in irritable bowel syndrome and its relationship with psychological factors. J Gastroenterol Hepatol 23, 1689–1694 (2008). 19120860 10.1111/j.1440-1746.2008.05574.x

[b20] Braak B. . Mucosal immune cell numbers and visceral sensitivity in patients with irritable bowel syndrome: is there any relationship? Am J Gastroenterol 107 (2012). 10.1038/ajg.2012.54 22488080

[b21] Kang C. S. . Extracellular vesicles derived from gut microbiota, especially Akkermansia muciniphila, protect the progression of dextran sulfate sodium-induced colitis. PLoS One 8, e76520 (2013). 24204633 10.1371/journal.pone.0076520 PMC3811976

[b22] Anhe F. F. . A polyphenol-rich cranberry extract protects from diet-induced obesity, insulin resistance and intestinal inflammation in association with increased Akkermansia spp. population in the gut microbiota of mice. Gut 64, 872–883 (2015). 25080446 10.1136/gutjnl-2014-307142

[b23] Holmen N., Isaksson S., Simren M., Sjovall H. & Ohman L. CD4+CD25+ regulatory T cells in irritable bowel syndrome patients. Neurogastroenterol Motil 19, 119–125 (2007). 17244166 10.1111/j.1365-2982.2006.00878.x

[b24] Willing B. P. . A pyrosequencing study in twins shows that gastrointestinal microbial profiles vary with inflammatory bowel disease phenotypes. Gastroenterology 139 (2010). 10.1053/j.gastro.2010.08.049 20816835

[b25] Wang, W. . Increased proportions of Bifidobacterium and the Lactobacillus group and loss of butyrate-producing bacteria in inflammatory bowel disease. J Clin Microbiol 52, 398–406 (2014). 24478468 10.1128/JCM.01500-13 PMC3911339

[b26] Malinen E. . Analysis of the fecal microbiota of irritable bowel syndrome patients and healthy controls with real-time PCR. Am J Gastroenterol 100, 373–382 (2005). 15667495 10.1111/j.1572-0241.2005.40312.x

[b27] Krogius-Kurikka L. . Microbial community analysis reveals high level phylogenetic alterations in the overall gastrointestinal microbiota of diarrhoea-predominant irritable bowel syndrome sufferers. BMC Gastroenterol 9, 95 (2009). 20015409 10.1186/1471-230X-9-95 PMC2807867

[b28] Lopez-Siles M. . Mucosa-associated Faecalibacterium prausnitzii and Escherichia coli co-abundance can distinguish Irritable Bowel Syndrome and Inflammatory Bowel Disease phenotypes. Int J Med Microbiol 304, 464–475 (2014). 24713205 10.1016/j.ijmm.2014.02.009

[b29] Vandeputte D. . Stool consistency is strongly associated with gut microbiota richness and composition, enterotypes and bacterial growth rates. Gut 65, 57–62 (2016). 26069274 10.1136/gutjnl-2015-309618 PMC4717365

[b30] Png C. W. . Mucolytic bacteria with increased prevalence in IBD mucosa augment *in vitro* utilization of mucin by other bacteria. Am J Gastroenterol 105, 2420–2428 (2010). 20648002 10.1038/ajg.2010.281

[b31] Vigsnaes L. K., Brynskov J., Steenholdt C., Wilcks A. & Licht T. R. Gram-negative bacteria account for main differences between faecal microbiota from patients with ulcerative colitis and healthy controls. Benef Microbes 3, 287–297 (2012). 22968374 10.3920/BM2012.0018

[b32] Sokol H. . Faecalibacterium prausnitzii is an anti-inflammatory commensal bacterium identified by gut microbiota analysis of Crohn disease patients. Proc Natl Acad Sci USA 105, 16731–16736 (2008). 18936492 10.1073/pnas.0804812105 PMC2575488

[b33] Reunanen J. . Akkermansia muciniphila Adheres to Enterocytes and Strengthens the Integrity of the Epithelial Cell Layer. Appl Environ Microbiol 81, 3655–3662 (2015). 25795669 10.1128/AEM.04050-14 PMC4421065

[b34] Derrien M., Vaughan E. E., Plugge C. M. & de Vos W. M. Akkermansia muciniphila gen. nov., sp. nov., a human intestinal mucin-degrading bacterium. Int J Syst Evol Microbiol 54, 1469–1476 (2004). 15388697 10.1099/ijs.0.02873-0

[b35] Everard A. . Cross-talk between Akkermansia muciniphila and intestinal epithelium controls diet-induced obesity. Proc Natl Acad Sci USA 110, 9066–9071 (2013). 23671105 10.1073/pnas.1219451110 PMC3670398

[b36] Dao M. C. . Akkermansia muciniphila and improved metabolic health during a dietary intervention in obesity: relationship with gut microbiome richness and ecology. Gut 65, 426–436 (2015). 26100928 10.1136/gutjnl-2014-308778

[b37] Masui R. . G protein-coupled receptor 43 moderates gut inflammation through cytokine regulation from mononuclear cells. Inflamm Bowel Dis 19, 2848–2856 (2013). 24141712 10.1097/01.MIB.0000435444.14860.ea

[b38] Kim M. H., Kang S. G., Park J. H., Yanagisawa M. & Kim C. H. Short-chain fatty acids activate GPR41 and GPR43 on intestinal epithelial cells to promote inflammatory responses in mice. Gastroenterology 145, 396–406 (2013). 23665276 10.1053/j.gastro.2013.04.056

[b39] Vivinus-Nebot M. . Functional bowel symptoms in quiescent inflammatory bowel diseases: role of epithelial barrier disruption and low-grade inflammation. Gut 63, 744–752 (2014). 23878165 10.1136/gutjnl-2012-304066

[b40] Kaser A., Zeissig S. & Blumberg R. S. Inflammatory bowel disease. Annu Rev Immunol 28, 573–621 (2010). 20192811 10.1146/annurev-immunol-030409-101225 PMC4620040

[b41] Vareille M. . Heme oxygenase-1 is a critical regulator of nitric oxide production in enterohemorrhagic Escherichia coli-infected human enterocytes. J Immunol 180, 5720–5726 (2008). 18390757 10.4049/jimmunol.180.8.5720

[b42] Robert C. & Bernalier-Donadille A. The cellulolytic microflora of the human colon: evidence of microcrystalline cellulose-degrading bacteria in methane-excreting subjects. FEMS Microbiol Ecol 46, 81–89 (2003). 19719585 10.1016/S0168-6496(03)00207-1

[b43] Hamamoto N. . Inhibition of dextran sulphate sodium (DSS)-induced colitis in mice by intracolonically administered antibodies against adhesion molecules endothelial leucocyte adhesion molecule-1 (ELAM-1) or intercellular adhesion molecule-1 (ICAM-1). Clin Exp Immunol 117, 462–468 (1999). 10469048 10.1046/j.1365-2249.1999.00985.x PMC1905382

[b44] Schloss P. D. . Introducing mothur: open-source, platform-independent, community-supported software for describing and comparing microbial communities. Appl Environ Microbiol 75, 7537–7541 (2009). 19801464 10.1128/AEM.01541-09 PMC2786419

[b45] DeSantis T. Z. . Greengenes, a chimera-checked 16S rRNA gene database and workbench compatible with ARB. Appl Environ Microbiol 72, 5069–5072 (2006). 16820507 10.1128/AEM.03006-05 PMC1489311

[b46] Edgar R. C., Haas B. J., Clemente J. C., Quince C. & Knight R. UCHIME improves sensitivity and speed of chimera detection. Bioinformatics 27, 2194–2200 (2011). 21700674 10.1093/bioinformatics/btr381 PMC3150044

[b47] Engel M. A. . Ulcerative colitis in AKR mice is attenuated by intraperitoneally administered anandamide. J Physiol Pharmacol 59, 673–689 (2008). 19212003

[b48] Wu H. G. . Regulatory mechanism of electroacupuncture in irritable bowel syndrome: preventing MC activation and decreasing SP VIP secretion. Dig Dis Sci 53, 1644–1651 (2008). 17999187 10.1007/s10620-007-0062-4

[b49] Viennois E., Chen F., Laroui H., Baker M. T. & Merlin D. Dextran sodium sulfate inhibits the activities of both polymerase and reverse transcriptase: lithium chloride purification, a rapid and efficient technique to purify RNA. BMC Res Notes 6, 360 (2013). 24010775 10.1186/1756-0500-6-360 PMC3847706

