# Supplementary material for: The human intestinal microbiota of constipated-predominant irritable bowel syndrome patients exhibits anti-inflammatory properties
Source: Sci Rep. 2016 Dec 16;6:39399. doi: 10.1038/srep39399 (PMC5159846; doi:10.1038/srep39399)
Supplement: Supplementary Information [file srep39399-s1.pdf]

The human intestinal microbiota of constipated-predominant irritable bowel syndrome patients exhibits anti-inflammatory properties

Alain P. Gobert[1,2,5]*, Giulia Sagrestani[1], Eve Delmas[1], Keith T. Wilson[2,3,4,5,6], Thomas G. Verriere[2], Michel Dapoigny[7], Christophe Del'homme[1], and Annick Bernalier-Donadille[1]*

[1]Institut National de la Recherche Agronomique (INRA), UR454 Microbiologie, Saint-Genès-Champanelle, France; [2]Division of Gastroenterology, Hepatology, and Nutrition, Department of Medicine, [3]Department of Pathology, Microbiology, and Immunology, [4]Department of Cancer Biology, [5]Center for Mucosal Inflammation and Cancer, Vanderbilt University Medical Center, Nashville, Tennessee, USA; [6]Veterans Affairs Tennessee Valley Healthcare System, Nashville, Tennessee, USA; [7]CHU Clermont-Ferrand, Service de Médecine Digestive et Hépatobiliaire, Hôpital Estaing, Clermont-Ferrand, France.

*Address correspondence to Alain P. Gobert, alain.p.gobert@vanderbilt.edu; Annick Bernalier-Donadille, annick.bernalier@clermont.inra.fr.

Supplementary Table S1. Richness and Diversity of the Fecal Microbiota

	Chao1 Richness Index	Shannon Diversity Index
Healthy Subjects	1579 ± 395	3.84 ± 0.19
C-IBS Patients	1922 ± 15	3.67 ± 0.002
N-HMAR	1997 ± 29	3.71 ± 0.005
C-IBS-HMAR	2099 ± 138	3.72 ± 0.24

Supplementary Table S2. Sequences of Primers for PCR

Species	Target gene	Sequence (5'–3')	Ref.
Eubacteria (Total bacteria)	16S rDNA	F: CGGTGAATACGTTCCCGG<br>R: TACGGCTACCTTGTTACGACTT	1
Bacteroides/Prevotella group	16S rDNA	F: CCTWCGATGGATAGGGGTT<br>R: CACGCTACTTGGCTGGTTCAG	1
Clostridium coccoides group	16S rDNA	F: CGGTACCTGACTAAGAAG<br>R: AGTTTYATTCTTGCGAAC	2
Clostridium leptum	16S rDNA	F: CACAATAAGTAATCCACC<br>R: CTTCCTCCGTTTTGTCAA	3
Roseburia/*Enterococcus rectale* group	16S rDNA	F: CGKACTAGAGTGTCGGAGG<br>R: AGTTTYATTCTTGCGAACG	4
Enterobacteriaceae	16S rDNA	F: CATTGACGTTACCCGCAGAAGAAGC<br>R: CTCTACGAGACTCAAGCTTGC	5
Bifidobacterium	16S rDNA	F: CGCGTCYGGTGTGAAAG<br>R: CCCCACATCCAGCATCCA	6
Faecalibacterium prausnitzii	16S rDNA	F: GGAGGAAGAAGGTCTTCGG<br>R: AATTCCGCCTACCTCTGCACT	7
Desulfovibrio sp.	16S rDNA	F: GGTACCTTCAAAGGAAGCAC<br>R: GGGCTTTCACCCCTGACTTA	8
Akkermansia muciniphila	16S rDNA	F: CAGCACGTGAAGGTGGGGAC<br>R: CCTTGCGGTTGGCTTCAGAT	7
Escherichia coli	16S rDNA	F: CATGCCGCGTGTATGAAGAA<br>R: CGGGTAACGTCAATGAGCAAA	This work
Mouse	*Ifng*	F: GCTTTGCAGCTCTTCCTCAT<br>R: GTCACCATCCTTTTGCCAGT	This work
Mouse	*Il1b*	F: ACCTGCTGGTGTGTGACGTTCC<br>R: GGGTCCGACAGCACGAGGCT	This work
Mouse	*Il17*	F: TCCAGAAGGCCCTCAGACTA<br>R: AGGACCAGGATCTCTTGCTG	This work
Mouse	*Nos2*	F: TCAGAGCCACAGTCCTCTTT<br>R: TCCATGCAGACAACCTTGGT	This work
Mouse	*Tnf*	F: GTAGCCCACGTCGTAGCAA<br>R: GGTGAGGAGCACGTAGTCG	This work
Rat	*Ifng*	F: CGAATCGCACCTGATCACTA<br>R: GACTCCTTTTCCGCTTCCTT	This work
Rat	*Il1b*	F: GCATCCAGCTTCAAATCTCA<br>R: ATCATCCCACGAGTCACAGA	This work
Rat	*Il6*	F: CCGGAGAGGAGACTTCACAG<br>R: CAGAATTGCCATTGCAACAAC	This work
Rat	*Il17*	F: GTGAAGGCAGCGGTACTCA<br>R: TTCTGGAGCTCGCTTTTGA	This work
Rat	*Il22*	F: GCCAGCCTTGCAGATAACA<br>R: ACTGGGGGAGCAGAACATC	This work
Rat	*Tnf*	F: GCCGATTTGCCATTTCATAC<br>R: TGGAAGACTCCTCCCAGGTA	This work
Rat/Mouse	*Actb*	F: CCAGAGCAAGAGAGGTATCC<br>R: CTGTGGTGGTGAAGCTGTAG	This work

1 Furet, J. P. *et al.* Comparative assessment of human and farm animal faecal microbiota using real-time quantitative PCR. *FEMS Microbiol Ecol* **68**, 351-362 (2009).

2 Malinen, E. *et al.* Analysis of the fecal microbiota of irritable bowel syndrome patients and healthy controls with real-time PCR. *Am J Gastroenterol* **100**, 373-382 (2005).

3 Matsuki, T. *et al.* Quantitative PCR with 16S rRNA-gene-targeted species-specific primers for analysis of human intestinal bifidobacteria. *Appl Environ Microbiol* **70**, 167-173 (2004).

4 Ramirez-Farias, C. *et al.* Effect of inulin on the human gut microbiota: stimulation of Bifidobacterium adolescentis and Faecalibacterium prausnitzii. *Br J Nutr* **101**, 541-550 (2009).

5 Bartosch, S., Fite, A., Macfarlane, G. T. & McMurdo, M. E. Characterization of bacterial communities in feces from healthy elderly volunteers and hospitalized elderly patients by using real-time PCR and effects of antibiotic treatment on the fecal microbiota. *Appl Environ Microbiol* **70**, 3575-3581 (2004).

6 Delroisse, J. M. *et al.* Quantification of Bifidobacterium spp. and Lactobacillus spp. in rat fecal samples by real-time PCR. *Microbiol Res* **163**, 663-670 (2008).

7 Collado, M. C., Derrien, M., Isolauri, E., de Vos, W. M. & Salminen, S. Intestinal integrity and Akkermansia muciniphila, a mucin-degrading member of the intestinal microbiota present in infants, adults, and the elderly. *Appl Environ Microbiol* **73**, 7767-7770 (2007).

8 Rinttila, T., Kassinen, A., Malinen, E., Krogius, L. & Palva, A. Development of an extensive set of 16S rDNA-targeted primers for quantification of pathogenic and indigenous bacteria in faecal samples by real-time PCR. *J Appl Microbiol* **97**, 1166-1177 (2004).

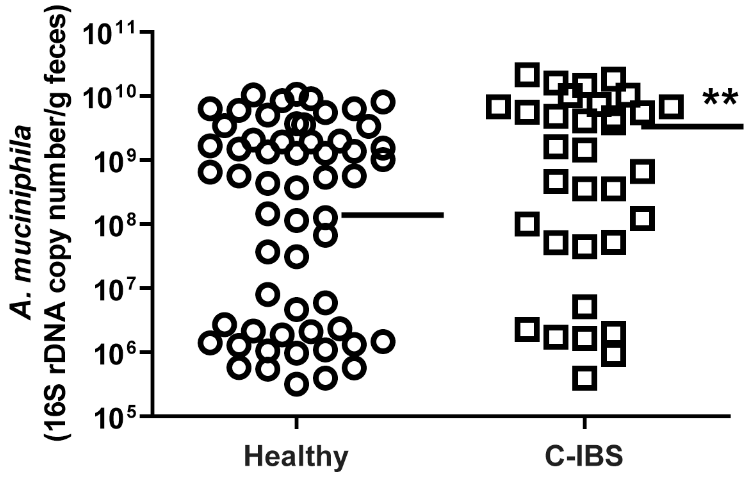

Supplementary Figure S1. Quantification of *A. muciniphila* in the feces of healthy individuals or C-IBS patients. Fecal samples from the subjects were collected under anaerobic conditions. Total DNA was extracted and the presence of *A. muciniphila* was quantified by real-time PCR. Each dot represents one individual. $^{**}P < 0.01$ vs. healthy subjects.

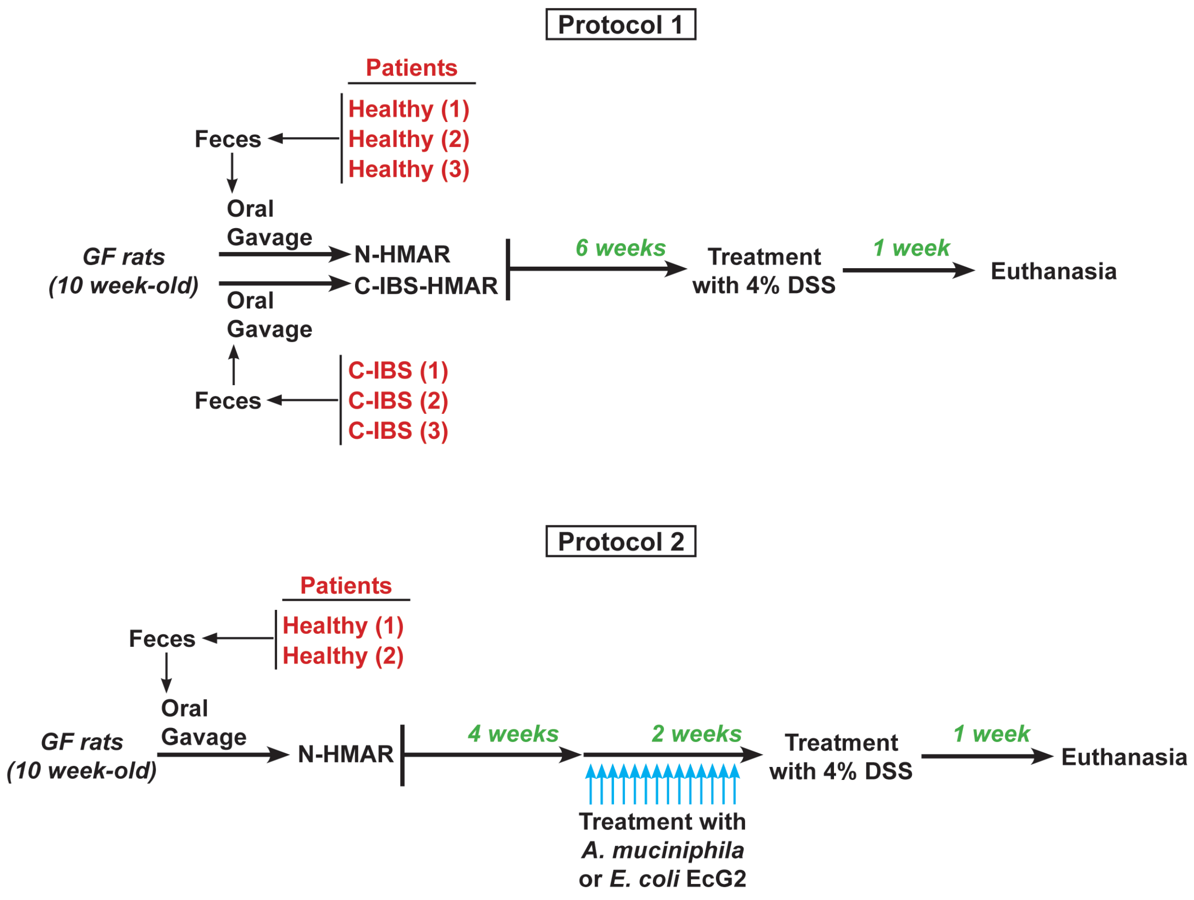

Supplementary Figure S2. Experimental design of the animal protocols used in this study.
